# Why do you shop there? A mixed methods study mapping household food shopping patterns onto weekly routines of black women

**DOI:** 10.1186/s12966-016-0333-6

**Published:** 2016-01-28

**Authors:** Katherine Isselmann DiSantis, Amy Hillier, Rio Holaday, Shiriki Kumanyika

**Affiliations:** Department of Community & Global Public Health, Arcadia University, College of Health Sciences, 450 S. Easton Road, 219 Brubaker Hall, Glenside, PA 19038-3295 USA; University of Pennsylvania, Perelman School of Medicine, Center for Clinical Epidemiology and Biostatistics, Philadelphia, PA USA; Department of City and Regional Planning, University of Pennsylvania, School of Design, Philadelphia, PA USA; University of Pennsylvania, Center for Public Health Initiatives, Philadelphia, PA USA; ChangeLab Solutions, Oakland, CA USA

**Keywords:** Obesity, Food environments, Health disparities, Black, Food access

## Abstract

**Background:**

The effectiveness of initiatives to increase healthy food access may be affected by where people decide to shop. People with poor neighborhood access to large grocery stores develop shopping patterns that require traveling to other areas, and some people who do have neighborhood access also travel elsewhere for food shopping. We sought to gain an understanding of household food shopping patterns in a sample of Black women in terms of where they shopped and why.

**Methods:**

All food shopping trips of 35 low- or middle/high-income black mothers or caregivers living with at least one child were identified from grocery shopping receipts collected over four consecutive weeks. Food shopping locations were mapped along with locations of participants’ homes and other places they visited during weekly routine travels (e.g. work, child’s school). Semi-structured individual interviews elicited narrative information about whether and how grocery shopping trips were linked to routine travels. Inductive content analysis was utilized to identify emergent themes from interviews. Themes were considered in relation to geospatial distances and travel patterns identified through mapping of participants’ shopping.

**Results:**

Participants shopped at an average of six different stores, traveling on average a total of 35 miles (*sd* = 41) (Euclidian distance) over the four weeks. The most frequented store was within a mile of home (57 %) or home or another place visited in the weekly routine for about 77 % of participants. Interview results emphasized the concept of convenience which referred to geographical proximity to the home or routine destinations and also to potential to save time because several stores were co-located or because the store layout was easy to navigate and familiar. Store selection also related to mode of transportation, pricing, and family preference for certain foods.

**Conclusion:**

People have specific reasons for consistently shopping in areas outside of their neighborhood of residence. Incorporating considerations other than proximity (e.g. time saving while shopping, promoting less familiar foods, pricing) into food environment interventions may facilitate use of new stores by neighborhood residents and thereby increase the viability of these stores as health-promoting food environment interventions.

## Background

In an effort to improve the dietary quality of US population segments which have limited access to healthy foods, public health researchers and practitioners have paid increasing attention to community food environments, i.e., the availability and types of food retail outlets within certain geographic areas [1]. Definitions of community food environments have typically used a shopper’s home address as the reference point for defining the geographical area relevant to food access. However, some research indicates that many urban food shoppers visit multiple stores and often bypass the closest stores [2–5], and that accounting for other locations visited during daily or weekly travel routines, also termed “activity space”, better defines a person’s food environment [5–7]. The latter research has begun to identify complex spatial patterns in food shopping. However, we know little about why shoppers develop these complex patterns.

Knowing what draws shoppers to particular locations can be helpful in designing public health initiatives aimed at improving dietary quality through environmental changes related to retail food access. We assessed food shopping patterns of black women shopping for their household with children in an urban area, with an emphasis on understanding where they shopped and why. The inclusion of only families with children was driven by an interest in early obesity prevention; greater availability of healthier foods in the home has been found to reduce obesity risk [8]. In urban areas, the availability of grocery stores is such that traveling farther for food shopping might not be expected but has been identified [2, 9]. The focus on black women was motivated by the higher than average prevalence of obesity and lower mean dietary quality scores among children and adults in black communities relative to US whites [10–12] and current efforts to improve access to healthy foods in the food retail environments of black &other underserved communities [13].

## Methods

### Study setting and participants

This analysis was conducted in data from a study in Philadelphia, Pennsylvania. Black women who self-identified as the primary family food shopper, had at least one child under the age of 18 years in the home, reported shopping at a supermarket at least once per month, and were willing to collect food receipts over four consecutive weeks were enrolled. Participants were recruited through flyers posted at supermarkets, other retail businesses, community centers, churches, non-profit agencies serving the community, and through word of mouth within 10 pre-selected zip codes with high proportions of black residents. These zip codes were within the same geographic subregion of the city, which was important because transportation resources (e.g. access to subways vs. buses, access to highways) and travel patterns differ by subregion. To account for the possibility that shopping might vary according to income or weight status, recruitment was designed to enroll approximately equal numbers of women with low versus middle or higher income levels and with body mass index (BMI) levels in the obese or non-obese range (i.e., BMI ≥ 30 or < 30, respectively). The sample size goal for this exploratory study was about 30 women; this number was chosen for feasibility, allowing for some attrition, sample diversity (mainly in terms of income level and obesity status), and in-depth analysis of household food shopping patterns.

Women were excluded from the study if they were pregnant, currently participating in a weight loss study, reported having a disease/condition which significantly impacted their food shopping patterns (e.g. Celiac disease, severe food allergies), or were intending to move out of the geographic study area within the four-week study period. Women participating in the Supplemental Nutrition Assistance Program (SNAP), the Supplemental Nutrition Program for Women, Infants, and Children (WIC), or both, were eligible although women who reported that it was “very” or “extremely difficult” to afford the food their family needed on a monthly basis were excluded.

### Procedure

Participants were screened by phone and enrolled in-person at a research office. Participation required three in-person meetings over a four-week period. During the enrollment meeting, participants were trained on food shopping receipt collection and were provided with receipt collection kits (plastic envelope to store receipts, log for items purchased without receipt or for free items received such as food pantry items). Reminders to collect receipts (through text or phone based on preference) were provided during the data collection period, where participants specified whether they preferred daily or weekly reminders. Participants submitted their family food shopping receipts at the mid-point and final meetings (two and four weeks after enrollment). They also completed a brief qualitative interview during the final meeting. Participants received a total of $175 in gift cards for their time and participation. All procedures were carried out in accordance with University of Pennsylvania’s human subjects’ protections and comply with the declaration of Helsinki and U.S. privacy regulations regarding individually identifiable health information (HIPAA).

### Data collection

#### Participant characteristics

Demographic and personal information was collected by self-administered questionnaire (e.g. height and weight, educational level (8^th^ grade; some high school; high school grad/equivalent, some college, college degree, some graduate school, graduate degree), marital status, age of children in the home, SNAP and WIC participation, and length of time living in current neighborhood). Income level was assessed with a question about all earned/unearned income for any persons over 15 years of age who contributed income to the household. Earned/unearned income and household size (number of adults and children) were used to categorize participants as falling above or equal to/less than the WIC income guidelines. Food Insecurity was assessed by the short form of the USDA Household Food Security Scale (6 items), also self-administered [14].

#### Shopping patterns & food purchase data

Study participants were asked to collect receipts from all purchases of food and non-alcoholic beverages over a four week period. These receipts were to be inclusive of family and personal food purchases from any store or restaurant type, including supermarkets, wholesale stores, small corner grocers, permanent/indoor and seasonal/outdoor farmers markets, meat markets, take-out restaurants, sit-down restaurants, and foods delivered to home. To get a complete picture of all food expenditures of the households, participants were asked to collect all food receipts- inclusive of non-prepared and prepared food items. For this analysis, only household food purchases were analyzed; prepared foods within a larger shopping trip were included. We did not include trips to restaurants or trips that only included prepared food items (e.g. lunch time purchase of pizza slice from prepared section of supermarket). This decision was made a priori, as we were most interested in the impact of shopping patterns on household food availability for families, not necessarily on individual level eating behaviors. At the mid-point and final meetings, a research staff member went through the receipts with the participant to review and clarify store names and locations and items purchased. Typical shopping practices, such as whether the women sometimes changed stores in order to take advantage of special prices, were assessed with an interview-administered questionnaire.

#### Weekly routine

Weekly routines were assessed through self-report, using an original “Mapping Everyday Life” interviewer-administered instrument. Participants were asked: *“take me through your regular week. We are interested in hearing about all the places you go throughout a typical week”.* They were told to report places they frequented at least every other week. They were given examples including work, child’s school, their own school, church, gym, community center, and child sporting events, but were reminded that their daily routines might not be limited to these examples. Participants provided a label for each place, such as “child’s school”, were asked to provide an exact or approximate address, describe the typical mode of transportation used to travel to this place, and whether travel to this place usually originated from home. Participants did not report the addresses of food store locations on the “Mapping Everyday Life” instrument; instead, food store addresses were obtained from the receipts. Home address was collected during the screening call.

#### Influences on store choice

**Fig. 1:**
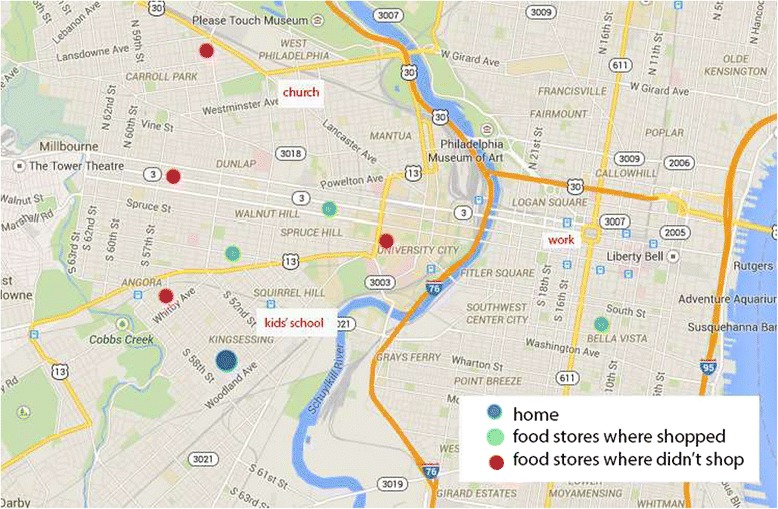
A map, with artificial data, similar to those shown to participants during map elicitation interviews is pictured. Participants had provided their routine travel destinations, such as work and children’s school, and the address. These locations were mapped along with their home, their actual food shopping locations (from receipts), and food stores that were not shopped at but which were near their home or routine travels. These maps were used to discuss reasons for shopping or not shopping at particular locations

At the final meeting, a semi-structured map elicitation interview was administered to assess the influence of food store proximity to home and/or routine travels on participants’ food shopping locations. Each participant was shown a map that marked her home, routine destinations, and food stores (Fig. 1; *note this example map utilizes artificial data to illustrate type of map shown to participants*). The food stores included those they had shopped at during the past four weeks and the major food stores in the area where they had not shopped but that were proximal to their home or routine destinations. After participants were oriented to the map and the locations charted on the map, they were asked a series of open-ended questions and encouraged to elaborate as needed. For each store on the map, they were asked “*Why do you shop there”? or “Do you know of this store? If so, why don’t you shop there”?* After participants described the various reasons for utilizing certain stores as opposed to others, they were directly asked about the importance of proximity in choosing where they shop for food: “*Thinking of all the reasons (which you have just described) why you shop at certain stores and avoid others, tell me how important a store’s closeness to your home or daily travels is”.* These qualitative interviews were 10–15 min in length and were audiotaped.

### Analytical plan

Data triangulation was performed using geospatial data, emergent themes from a qualitative content analysis of map elicitation interviews with participants, and the mapping of participant shopping patterns and routines to illustrate themes identified through the content analysis [15]. The objective was to add validity to the study findings by analyzing the research question utilizing multiple analytical and data collection methods.

Spatial analyses were performed to describe food shopping trip characteristics, including locations relative to home and routine destinations. Data on locations of each participant’s home, potential supermarket shopping locations (based on a database of the supermarkets in the study area which had been developed for a related study), actual food shopping locations, and locations of routine destinations were entered into a data base and geocoded with ArcGIS 10.0. The geocoded data were used to calculate the Euclidean distance from the participant’s home address to each food store using the XY to Line tool. Euclidean distance from participant’s home to the most frequented food store over the four week period was calculated to assess the most typical distance to the store from home. A variable was created to indicate whether the participant’s most frequented supermarket was not the one closest to their home.

Results of the map elicitation interviews were transcribed, coded using NVivo software, and analyzed qualitatively. The goal of the content analysis was to develop themes that provide context for shopping patterns identified in participant maps. Themes emerged from the coded text, where coded text was read and coded inductively. The constant comparative method [16] was utilized in order to make differences within and between themes apparent.

Individual participant maps developed in ArcGIS 10.0 were reviewed by three members of the research team [KID, AH, SK] in order to assess, qualitatively, the apparent relationships of food shopping locations to home and routine destinations and to identify any characteristic patterns or commonalities among participants. From this review, individual shopping patterns of participants’ which illustrated elements from emergent qualitative themes related to travel patterns were identified to provide a prime example illustration, and are included in the theme description. Maps of individual shopping patterns are not shown in order to reduce the risk of participant identification.

## Results

### Participant characteristics

Table 1 shows characteristics of the 35 out of 37 enrolled women who were included in analyses; one participant did not complete the four weeks of receipt collection and one participant was excluded because of a very high number of receipts, relative to the rest of the sample. The stratified recruitment strategy was effective in enrolling approximately equal proportions of women with or without BMI levels in the obese range. Women with incomes below the WIC cut-off comprised less than half of the sample, but slightly more than half of the women were SNAP participants. Half of the women were college graduates. As required for eligibility, all participants had at least 1 child under age 18 in the household; 94 % had a child younger than 10 years of age at home.

**Table 1:** Description of participants (*n* = 35)

Variable	Mean (sd)^a^
Age	38.9 (11.1)
Household Income above WIC eligibility cut-off	13 (37 %)
BMI ≥ 30 (obese)	19 (54 %)
Employed (Full- or Part-time)	21 (60 %)
College educated	18 (51 %)
Married	14 (40 %)
Children 10 years and younger in household	33 (94 %)
Number of persons in household	3.8 (1.8)
Participant in SNAP program	21 (60 %)
High/Marginal Food Security^b^	18 (51 %)
Use car to food shop^c^	18 (51 %)

^a^or N (%) where specified

^b^This variable was created based on the USDA Six-item Short Form Food Security Scale

^c^This variable was created based on the reported method of travel on the majority of shopping trip

### Quantitative description of shopping patterns

Table 2 provides quantitative data on shopping patterns. On average, participants made 13 trips to 6 different stores over four weeks. The most frequented store was an average of 1.8 miles from participants’ homes. The most frequented store was within a mile of home for about half of participants (57 %) and was within a mile of either home or a routine destination visited in the weekly routine for 77 %. Only 14 % (*n* = 5) of participants shopped exclusively within 1 mile of their home and 11 % (*n* = 4) never shopped within 1 mile of their home (not shown).

**Table 2:** Descriptive data on participants’ food shopping patterns over 4 weeks period

Variable	Mean (SD)^a^
Average number of food shopping trips^b^	15.22 (11.8)
Average number of stores used^b^	6 (3.0)
Average amount spent per household over^b^	$407.08 ($222.34)
Usual mode of travel to food shop^c^	
Car (own car or riding with someone else)	22 (63 %)
Walking	9 (26 %)
Public transportation	5 (14 %)
Average sum of travel for all food shopping^d^ (mi)	34.9 (40.5)
Average distance to most frequented store^d^ (mi)	1.8 (2.3)
Most frequented store was supermarket/grocery	27 (77 %)
Most frequented store within 1 mile from home	20 (57 %)
Most frequented store within 1 mile from home or routine destination	27 (77 %)

^a^or N (%) where specified

^b^based on mean of individual shopper averages over the four week period

^c^Usual travel mode was determined by asking typical mode of travel to most frequented store

^d^estimated based on Euclidian distances from home location

### Explanations for shopping locations

Themes emerging from the map elicitation interviews related primarily to convenience. Convenience encompassed store proximity to home and other routine destinations and also highlights other factors that impacted perceived convenience. Convenience was also balanced with other factors such as store pricing and child-friendly shopping environments.

#### Stores which are close to home or routine destination

The primary theme was related to the importance of proximity to a home or a routine destination as a matter of convenience. Convenience of a location included issues related to time-saving and ease of access. Some participants preferred shopping close to home. Their explanations for shopping close to home often involved limitations (e.g. family responsibilities, lack of transportation, and mobility limitations) which kept them from having the option to shop further from home. For example, this shopper explains why she shops close to home: ***“It is very important to me, the closeness of the supermarket. Because I have an elderly mother at home. My time period for me to get from work to point A is very limited”.*** Participants spoke of knowing their daily travel pathways and often attempted to complete errands, including food shopping, along established, familiar routes. One participant stated: ***“We are dropping our child off at a certain activity I will stop at the store, you go on the errand. You work it into your schedule, for scheduling purposes it is more convenient if it is close by along your travelling path”.*** In these cases convenience had less to do with distance from home and more to do with being situated near homes of relatives, work places, child activity locations, or along other time-saving routes traveled for other reasons. For example, one participant completed all of her food shopping far from home but close to her church. She noted that the stores near her church offer convenience: **“I shop there only for convenience because I’m in that vicinity”,** but also said that she would shop near home if she felt the stores were as “bright” and “clean” as the stores near church. She stated **“you want something in your community where you can frequent”.** Thus, the perceived quality of the stores near her home was an additional factor in her decision to shop close to a routine destination outside of her home neighborhood.

A pattern of shopping along established pathways near either home or other routine destinations was observed across the sample. For example, one participant shopped at 10 stores over four weeks; 4 stores were within 1.0 Euclidian mile from home. All of the remaining stores were within 1 mile of a routine destination. This participant, who did not have access to a car, also related shopping along pathways to public transportation routes. For example, she described a particular store on a public transportation route to be the most convenient during the work week, stating ***“…the way I go home is the El [subway]. So, if I’m thinking of a meal to make before I get home, I’ll stop at [Store X] and then come back and get on the 40 [bus]”.***

#### Convenience of shopping areas with a variety of stores

Participants also discussed some shopping trips that were based on the ability to shop at numerous stores, including food and non-food stores. Participants described utilizing multiple stores to obtain the preferred items. For example, when a participant was asked why she shopped at a chain grocery store location that was further from her home, she said ***“when I go to that location to just hit [Food Store A] right afterward, because [Food Store A] and [Food Store B] are in close proximity”.*** These types of shopping trips were often described as “shopping events”, with one participant saying, ***“I do the big shopping”*** on these days***.*** This shopping pattern was illustrated by a participant who shopped at two supermarkets within 1 mile of each other; this cluster of stores was about 8 miles from her home. Participants also described traveling to a location to access multiple food and non-food stores. One participant’s statement exemplified this: ***“Because [neighborhood name] has a lot more stores…right within the area. You have [Supermarket A], you have [Supermarket B], I go to [Pet Supply Store], I go to [Big Box Retailer], I go to [Home Improvement Store]…so I have all those stores right here. And sometimes, even in the midst of me shopping, I have got the gas station right there…so I prefer to go in an area where I have more of a variety, even though it is further”.***

#### Time-saving inside the store

In the process of assessing the importance of store proximity to home or routine travels, participants also described aspects of convenience which were not defined by proximity to home/routine locations. These aspects of convenience included being familiar with a store’s layout, efficient customer service, and family-friendly qualities of stores. For example, when comparing reasons for traveling to a further store, one participant said: ***“it just seems like I can find stuff a lot better [in Store A]. So I go to a store, not just for what they are selling, but also for convenience and how I feel in the store”***. Store layouts were important because being familiar with a store saved time. A participant described familiar layouts: ***“The layout, I know where everything is, boom-boom-boom. I can go in and I am in and out”.*** Beyond choosing familiar and preferred stores, participants described avoiding stores that make food shopping trips longer than necessary because of crowding and/or poor service. Also, conveniences like parking lots and stores located in low traffic areas were connected with saving time by participants. Participants also spoke of needing particular items for their family, and that a store where you could buy “everything” the family needed was convenient, even if it meant you had to travel further. When asked what she looks for in a food store, one shopper said: ***“Having what I want, having what’s on my list. I don’t want to go to a store that’s close only to be able to get two things”.***

#### Balancing multiple responsibilities

This sample of female household food shoppers consistently described having multiple responsibilities in their lives. These responsibilities provide an important context for interpretation of their food shopping choices. In some cases, the responsibility of managing household finances leads them to prioritize price when food shopping. But they also have limited time and must meet responsibilities at work and care for children. They might also be responsible for older relatives, and even helping neighbors with food shopping. For example, one woman spoke of the challenges of finding time in her schedule in order to take advantage of sale prices: ***“With schedules as children get older, I have to work into more of a time factor. I really wish I could get back to…spending more time at [Supermarket] and look at some of their sales more often”.*** When speaking about their food shopping patterns, participants described how these responsibilities drive them to make certain choices, which often means that shopping preferences, including where they shop, are secondary to fulfilling family responsibilities.

#### Shopping with children

Mothers/caregivers also spoke of picking shopping options which worked when children were with them. Many women spoke of the challenges of shopping with children in tow and described aspects of stores which made this more feasible. Some women spoke of the trip to the store, emphasizing the need to limit the travel time: ***“When I had two kids, it was hard getting them ready, you know back and forth from the store in the car, you know. Even without a car, it just .. period – it has to be close. It makes it easier”.*** Others talked of in-store amenities which can improve the shopping experience such as having bathrooms that children can use or having carts designed for children (e.g. car attached to front).

#### Influence of proximity while considering price

During the course of the map elicitation interviews, participants described a give-and-take between location-based convenience and food prices in their store choice decisions. Participants often talk about balancing the proximity of the store with the potential monetary savings associated with going to a store farther away with better prices; both regular and sale prices were considered. For example, one participant stated ***“It would make sense to me to go out of my way for $1.50 less, so I might as well go there and get what I need …My priorities are convenience and price. They sometimes switch numbers where one takes priority over the other, but it is convenience and price”.*** In relation to traveling further when seeking low prices, some participants described how “good prices” draw them to certain stores, even if it takes them out of their way. Thus, the give-and-take between price, convenience (in the sense of proximity), and a shopper’s willingness to travel involved conscious choices of whether to travel further for “good prices” or choose more conveniently located stores and pay higher prices.

## Discussion

Thus far, environmental interventions targeting supermarkets have focused primarily on location, including policies that provide financial incentives for opening new supermarkets in neighborhoods designated as food deserts [17]. Although not always explicit, the presumed associated rationale for improving dietary quality with these interventions is that: (1) these stores will make it more convenient for residents to access a large grocery store that sells healthy products and they will, therefore, shop at this location; and (2) shopping at the new store will lead to purchasing a healthier mix of products. The latter assumption is supported by some cross-sectional studies reporting correlations between dietary quality or BMI with access to supermarkets [18–20]. Linking improvements in an individual’s dietary quality or BMI to the presence of a new supermarket has been more difficult, although other positive impacts of new supermarkets on neighborhoods (e.g. improved perceived food access, positive economic impact) have been identified [13, 21]. Moreover, it is not likely that one food store will address all purchasing behaviors in a family, as it has been found that multiple stores are often utilized to make household food purchases [22]. For example, shoppers in this study utilized 6 stores across the four week observation, on average, which might mean the assumption that people are depending one store is arcane.

The null results of past studies with respect to the effects of new supermarkets on individual dietary quality may relate to a lack of validity of the fundamental assumption that neighborhood residents will shop at the closet store. In the current study, the spatial data illustrated that this urban sample of black, low- and middle/high-income women have high mobility, traveling beyond their immediate neighborhood for food shopping, even though none of the participants lived in areas designated as food deserts. Only about half of these women shopped most frequently near their homes and very few shopped exclusively in their home neighborhoods. This is consistent with past findings that shoppers’ community food environments include locations along their routine pathways rather than only areas near their homes [5–7]. In addition, our findings add insights about participants who did not shop close to either home or their routine destinations; this applied to 25 % of the women in our sample.

Our qualitative findings about *why* food shoppers develop such patterns are potentially useful for informing future interventions that manipulate physical food availability in communities. Although it is not surprising that these shoppers described convenience as being paramount when determining where they shopped, the complexity of how convenience was defined was revealing. Convenience was not exclusively described as related to a store’s proximity to home neighborhood. It included assessments of time saving aspects afforded by in-store characteristics offered and was described as being weighed against availability of preferred family foods and prices offered in stores, as well as considering the need to manage multiple responsibilities (e.g. is it on the way to child’s sports practice?). Time saving itself had several dimensions, e.g., alignment with travel routines, shopping along routine pathways, shopping in familiar stores where the layout is known, and shopping in areas where multiple stores could be accessed. Other researchers have reported that time constraints are a barrier to achieving healthy diets [23–25]. These findings suggest shoppers proactively navigate their environments in order to cope with these constraints; thus, parsing out which factors take priority when choosing a store is difficult.

Prior published research also shows that price is an important determinant of what food shoppers purchase [2, 5]. This may affect decisions about where to shop as well as what to buy once in the store. The mention by some women of circumstances in which good prices would be sufficient to offset the time needed to travel further suggests that prices influence store choice in combination with time and distance factors.

Taken together, the spatial analysis and the qualitative interview results suggest that public health initiatives aiming to improve food environments by increasing availability of retail outlets in a particular geographic area may not increase perceived convenience for residents of the area solely on the basis of location [2, 3, 26, 27]. Addressing other aspects related to shoppers’ need for convenience, including time saving within the store, as well as price and product preferences, may increase the likelihood that more area residents will patronize new stores and shop there more often, even if they continue to also shop elsewhere.

The strengths of the current study include the objective measure of food shopping locations (from receipts), rather than self-reported measures, and the use of mixed methods to acquire depth in understanding why shopping occurs where it does. The focus on black women shopping for their household is an additional strength in that is provides insights about an understudied, high-risk consumer population that is of interest for targeted and tailored public health interventions. In addition, having an approximately equal mix of obese and non-obese and low and middle/higher income come increases the potential generality of insights obtained. We readily acknowledge that caution is needed when drawing generalities based on a small sample. In addition, we recruited women who shopped at a supermarket at least once per month and none happened to live in an area designated as a food desert. We have no way of knowing how different shopping locations are for women who use supermarkets less often or whether there are other key influences on location of stores used that did not emerge. In particular, although our underlying interest was in pathways of access to healthy foods, we did not directly query or probe this issue. Thus, we cannot answer the question of whether or how geographical shopping patterns of black women who vary on the motivation to obtain healthy foods might differ. Last, while the method of map elicitations was useful for stimulating the discussion about reasons for shopping or not shopping at particular locations, it might have drawn more attention to geospatial patterns of the participants than if participants had been interviewed without maps.

## Conclusions

Food access interventions are an essential strategy for addressing the obesity-related health disparities experienced by black Americans. Proximity of a store to one’s home or activity space held importance for many of these low- and middle/high-income black shoppers, but convenience, time saving potential (while shopping), pricing, and family preference for certain foods were influential factors as well. This study also further illustrated that shoppers use multiple shopping locations to make their household food purchases. The current findings highlight the importance of acknowledging this use of multiple stores as well as incorporating other aspects of shopping decisions (e.g. price, family preferences) into food environment interventions, particularly those oriented to black family food shoppers living in urban areas. This would facilitate the viability of new stores from a business and overall neighborhood enhancement perspective. This would also facilitate greater exposure of neighborhood residents to any special health-oriented in-store marketing undertaken in new neighborhood stores that have been developed through public health partnerships.

## Abbreviations

BMI: body mass index
SNAP: supplemental nutrition assistance program
WIC: the special supplemental nutrition program for women, infants, and children
USDA: United States Department of Agriculture
